# Social pain: A systematic review on interventions

**DOI:** 10.12688/f1000research.159561.1

**Published:** 2025-01-10

**Authors:** Brandon M. Brooks, Francisco J. Cordero, Stephen L. Alchermes, Bradley M. Brooks

**Affiliations:** 1 Surgery, Columbia VA Health Care System, Columbia, SC, USA; 2 The University of Texas at Austin, Austin, Texas, USA; 3 Psychiatry, AltaPointe Health Systems Inc, Mobile, Alabama, USA

**Keywords:** social pain, rejection, emotional distress, pain

## Abstract

Social pain is emotional distress caused by harm or threat to social connections that results in social exclusion, rejection, or loss. Social Pain is also a potentiator of physical pain. Supportive social relationships are widely recognized for their impact on maintaining health and well-being. The Passion of Jesus Christ serves as a quintessential example of social pain (i.e., desertion, betrayal, denial) potentiating physical pain (i.e., beatings, Crown of Thorns, crucifixion). Christ opts to forgive. Although forgiveness is one solution to reduce social pain, other interventions exist. This review seeks to identify and summarize interventions associated with reducing social pain. We conducted a systematic review using Medline (PubMed), Google Scholar, and Cochrane CENTRAL to identify relevant articles. Results: The database searches produced 548 articles. Fourteen randomized controlled trials (RCTs) were included in this systematic review. Acetaminophen, both deceptive and open-label placebos, mindfulness training, and psilocybin were found to reduce social pain. Of note, the combination of acetaminophen and forgiveness yielded superior results compared to either acetaminophen or forgiveness alone. Pharmacological interventions operate on the premise that the neural pathways responsible for physical pain also play a role in social pain. Both pharmacological and non-pharmacological interventions are available for reducing social pain.

## 1. Introduction

There is growing recognition in the literature of the strong association between social pain, also referred to as social rejection pain, and the exacerbation of physical pain ( Zhang, Zhang, & Kong, 2020; Fairhurst et al., 2012; Eisenberger, Lieberman, & Williams, 2003). Studies have indicated that social pain can potentiate physical pain, leading to heightened sensitivity and increased discomfort ( Meyer, Williams, & Eisenberger, 2015; Hawkley & Cacioppo, 2010; Kross et al., 2011). Social pain is psychological pain or emotional distress caused by perceived harm or threat to social connections that results in social exclusion, rejection, or loss ( Eisenberger, 2015). Building and maintaining close social connections is essential for human survival and has been recognized as a fundamental human drive ( Baumeister & Leary, 1995). When these crucial social bonds are threatened, individuals may experience a range of negative emotions ( Leary & Springer, 2001). This emotional state is consistently associated with heightened brain activity, particularly in the anterior cingulate cortex (ACC) ( Rotge et al., 2014), but also in the insula, the inferior orbitofrontal cortex (OFC), and the middle frontal gyrus (MFG) ( Cacioppo et al., 2013; Eisenberger, Lieberman, & Williams, 2003).

Positive and supportive social relationships play a crucial role in maintaining health and well-being, serving as a protective factor against the effects of social stress ( Broadhead et al., 1983; Cohen & Wills, 1985). Research has consistently shown that social support has positive effects on the cardiovascular, endocrine, and immune systems ( Uchino, Cacioppo, & Kiecolt-Glaser, 1996). On the other hand, experiences of social isolation, exclusion, or rejection can have profound negative consequences. Lack of social support and increased social isolation are associated with a higher risk of mortality, comparable to risk factors such as smoking, obesity, and high blood pressure ( Uchino, 2006). The impact of rejection sensitivity may vary among different groups. For example, individuals suffering from borderline personality disorder show higher levels of rejection sensitivity compared to both healthy individuals and other clinical groups, such as those with social anxiety disorder ( Bungert et al., 2015; Staebler et al., 2010). Prolonged social rejection is reported to increase the likelihood of psychosomatic and other psychosocial issues such as antisocial behaviors, depression, anxiety, feelings of alienation, and suicidal tendencies ( Bungert et al., 2015; Staebler et al., 2010; Uchino, 2006; Jacobs et al., 2000).

The Passion of Jesus Christ highlights quintessential social pain prior to immense physical pain and death. At the Last Suppler, Luke 2:21 accounts that Jesus announced to His disciples that “And yet behold, the hand of the one who is to betray me is with me on the table.” Betrayal is a type of rejection that is often found at the heart of social pain. Jesus not only suffers rejection by Judas Iscariot, but also by his closest friends when they fall asleep in the Garden of Gethsemane. Additionally, Simon-Peter goes on to deny Jesus three times. The crowds, made up largely of Jesus’s own people (“His Tribe”) don’t call for his freedom but instead ask the Roman governor to release a convicted insurrectionist and murderer. Such social pain very well may have been traumatic and worsened Jesus’s physical pain. Interestingly, Jesus offers forgiveness during His crucifixion. Forgiveness is a powerful yet challenging potential solution to alleviate solution pain because it involves intense vulnerability.

Researchers have explored various strategies to mitigate the negative impacts of social pain, including non-pharmacological interventions, such as forgiveness and mindfulness, and pharmacological approaches ( Henningsson et al., 2021; Smith, 2009; Toussaint et al., 2010). One pharmacological approach involves the use of oxytocin, a hormone implicated in human social bonding ( Henningsson et al., 2021). Studies have shown that plasma oxytocin levels increase during sexual activity ( Carmichael et al., 1987), and genetic variations in the oxytocin receptor gene (OXTR) have been linked to the quality of pair bonds ( Walum et al., 2012). Research suggests that the impact of intranasal oxytocin on social cognition is influenced by individual differences, such as gender, as well as the social setting ( Bartz et al., 2011; Di Simplicio & Harmer, 2016; Olff et al., 2013). Another drug, acetaminophen, typically used to treat physical pain, has also shown effects on social pain. Although the precise mechanisms by which acetaminophen affects physical pain are not entirely clear, it seems to influence central nervous system pathways that play a role in both physical and social pain ( Smith, 2009; Toussaint et al., 2010). Research has demonstrated that acetaminophen diminishes individuals’ emotional responses to both social and physical pain experienced by others ( Mischkowski, Crocker, & Way, 2016). This systematic review aims to explore some of the interventions that reduce social pain.

## 2. Methods

This systematic review was carried out following guidelines published in the Preferred Reporting Items for Systematic Reviews and Meta-Analyses (PRISMA) statement 2020 ( Page et al., 2021). *We used* Zenodo.org *as an online repository (CC0 license) with the following DOI (* doi.org/10.5281/zenodo.14559893 *) for our Prisma checklist and flow diagram.*

### 2.1 Information sources

Three databases were used for the literature search. These were PubMed, Google Scholar, and Cochrane Central Register of Controlled Trials (CENTRAL). Articles were searched from database inception on March 1 ^st^, 2024. The keywords in PubMed were limited to only those appearing in the title and abstract of the study. The article search was also filtered to include clinical trials only. No filters were applied in either CENTRAL or Google Scholar. The search results were then exported and subjected to a thorough study selection process ( Table 1).

**Table 1.** Search terms.

Database	Search terminology
PubMed	(“social pain”[Title/Abstract] OR “rejection pain”[Title/Abstract] OR “social exclusion”[Title/Abstract] OR “social rejection”[Title/Abstract] OR “hurt feelings”[Title/Abstract])
Google Scholar	(“social pain” OR “rejection pain” OR “social exclusion” OR “social rejection” OR “hurt feelings”)
CENTRAL	(“social pain” OR “rejection pain” OR “social exclusion” OR “social rejection” OR “hurt feelings”)

### 2.2 Eligibility criteria

Only randomized clinical Trials (RCTs), written in English, and including only a human population were included in this review. The aim of the RCT had to be to investigate the effect of a certain intervention on the reduction of social pain. The inclusion criteria are based on the following PICO format: our population of interest were humans. We looked at all interventions aimed at reducing social pain. Our control group was not limited. Our outcome of interest was any impact of social pain. All three authors had to agree that the outcome dealt with social pain via abstract review and full review of the article.

## 3. Results

### 3.1 Search results

**Figure 1.**
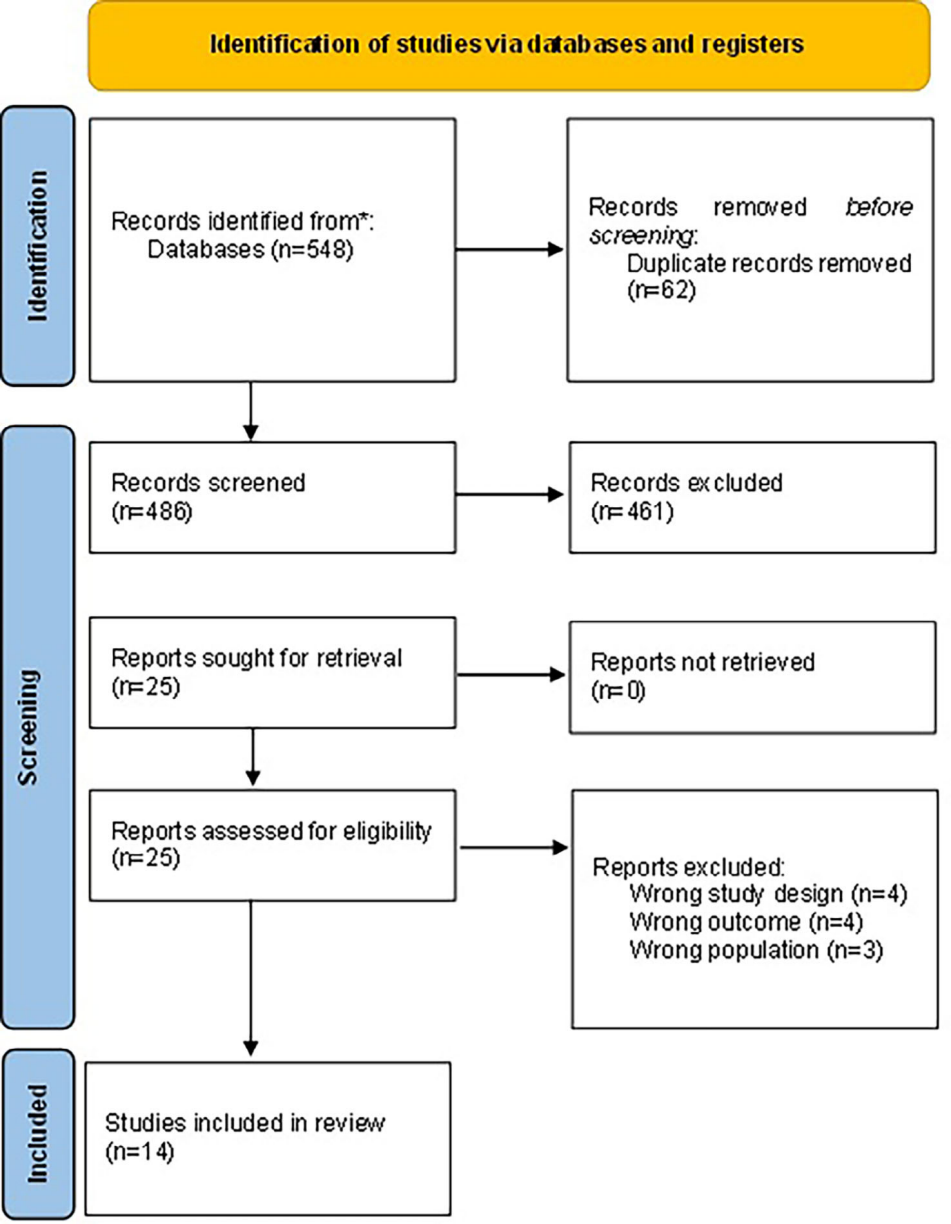
PRISMA flow diagram. An overview of the study selection process for this systematic review on the interventions for Social Pain.

The database yielded a total of 548 articles ( Figure 1), which included 139 from PubMed, 309 from CENTRAL, and 100 from the first 10 pages of Google Scholar. 62 studies were excluded as duplicates, and 461 were excluded during the title and abstract screening. Eleven studies were further excluded during the full-text review for reasons outlined in the flowchart. Ultimately, 14 studies, all of which were randomized controlled trials (RCTs), were included in this review ( Table 2).

**Table 2.** Results of Data Extraction.

Study	Participants	Experiment	Control	Outcome measure
Alvares, Hickie, and Guastella (2010)	74 healthy participants, 37 men, mean age of 21.91± 5.93 years	oxytocin (n = 35)	placebo (n = 39)	Fundamental Needs Questionnaire
Dewall et al. (2010)	62 healthy participants	1,000 mg daily of acetaminophen (n = 30)	placebo (n = 32)	Hurt Feelings Scale
Ghavidel-Parsa et al. (2021)	71 fibromyalgia patients	Duloxetine (n=44, mean age of 44.84±9.38 years)	Pregabalin (n=27, mean age of 46.63±7.77 years)	Illness Invalidation Inventory (III), Beck Depression Inventory-II (BDI-II)
Henningsson et al. (2021)	100 participants, 50 male, mean age of 23 ± 4 years	oxytocin (n=50)	placebo (n=50)	VAS scale
Hofman, Wieser, and van der Veen (2021)	72 healthy participants, 24 males, mean age of 21.78±3.57 years	1,000 mg daily of acetaminophen (n=33, 30.6% male, mean age of 20.1 years)	placebo (n=36, 36.1% male, mean age of 22.5 years)	Beck Depression Inventory (BDI-II)
Keng and Tan (2018)	118 participants with high borderline personality (BPD) traits, 36% male, mean age of 21.71± 2.70 years	brief mindfulness and loving-kindness meditation (LKM) (n=79)	no-instruction control (n=39)	5 -item VAS
Koban et al. (2017)	40 right-handed participants, 19 males, mean age of 20.8 years	placebo (n = 20)	control (n = 20)	5 -item VAS
Miller et al. (2014)	48 healthy participants, 16 males, mean age of 20.69 ± 2.17 years	25 g of glucose (n=24)	sucralose placebo (n=24)	5-point scale
Mohr, Kirsch, and Fotopoulou (2017)	93 healthy, right-handed students, 100% female, mean age of 23.13± 2.63 years	oxytocin	placebo	self-reported feelings of rejection
Petereit et al. (2019), Preller et al. (2016)	21 healthy volunteers, 12 males, mean age of 26.48 ± 4.76 years	psilocybin (0.215 mg/kg) (n=21)	placebo, maltose (n=21)	Positive and Negative Affect Schedule (PANAS)
Radke et al. (2020)	43 healthy participants, 100% female, mean age of 22.8 ± 3.1 years	oxytocin (n=22)	placebo (n=21)	self-reported feelings of rejection
Slavich et al. (2019)	42 healthy adults, mean age of 19.48± 1.27 years	1,000 mg of acetaminophen daily (n = 15)	placebo (n = 14) or control i.e. no-pills (n = 13)	Hurt Feelings Scale (HFS), Offense-Specific Forgiveness Measure (OSFM)
Stumpp et al. (2023)	74 participants, 51 females, mean ag *e of* 27.27± 11.64 years	placebo (n=38)	no-treatment (n = 36)	9-point Likert-type item
Zhang et al. (2021)	61 healthy participants	oxytocin (n=30)	placebo (n=31)	self-reported pleasantness

* Please note that Petereit et al. (2019) was a follow-up study to Preller et al. (2016). They both reported the same clinical trial.

### 3.2 Results of quality assessment

**Figure 2.**
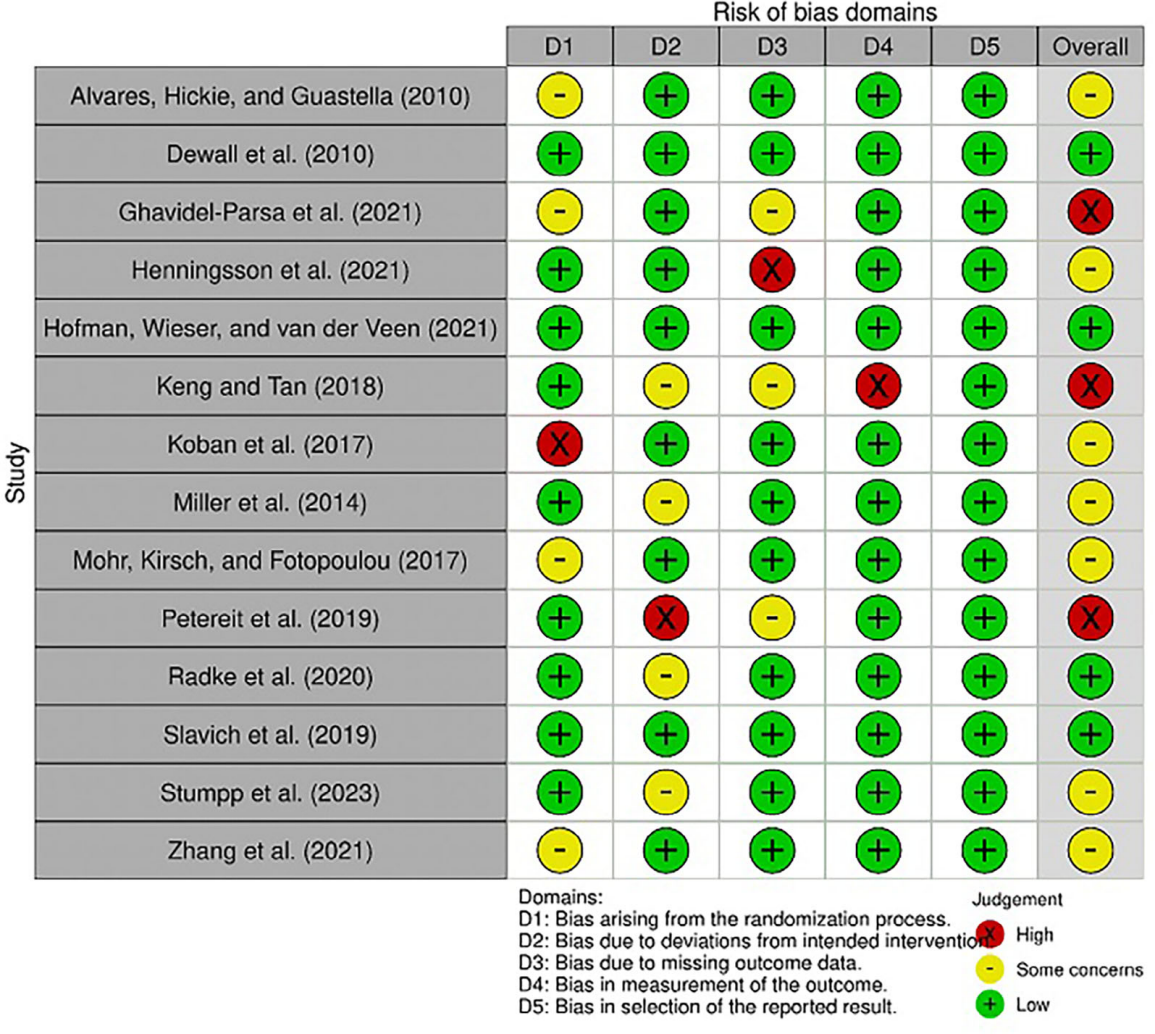
Risk of bias graph. D1: Risk of bias arising from the randomization process, D2: Risk of bias due to deviations from the intended interventions (effect of assignment to intervention), D3: Missing outcome data, D4: Risk of bias in measurement of the outcome; D5: Risk of bias in selection of the reported result.

**Figure 3.**
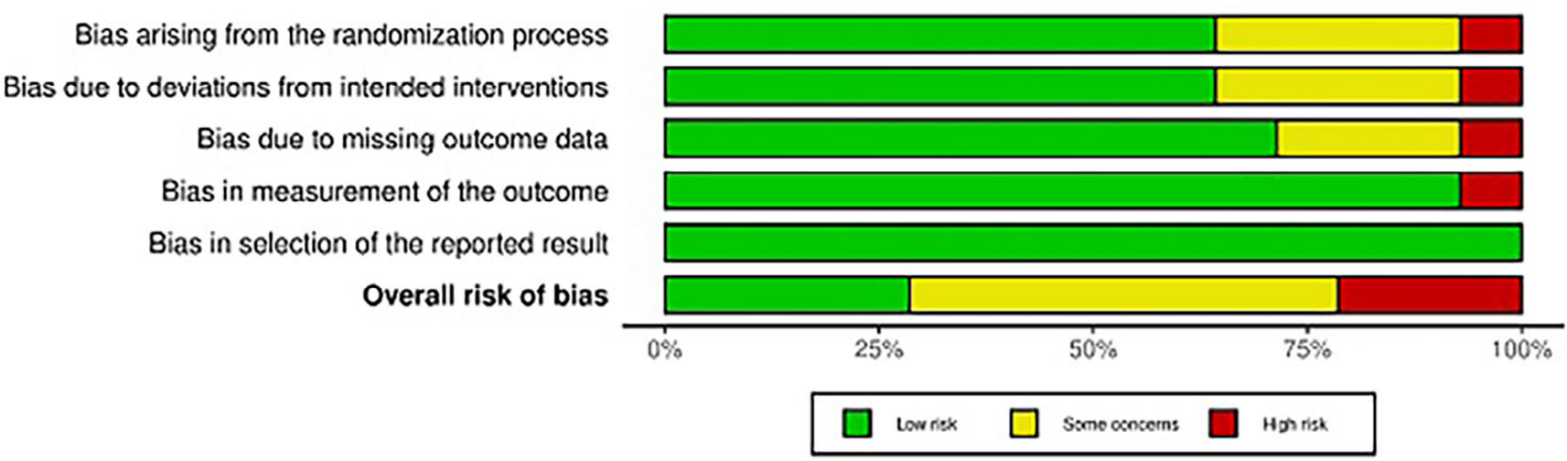
Risk of bias summary. A depiction of the bias in the included studies.

Most of the studies exhibited some concerns regarding study bias, with only three classified as having a high risk of bias, while four were considered to have a low risk of bias ( Figure 2). The domains of randomization and deviation from intended interventions were areas of concern. Overall, the studies demonstrated a low risk of bias with some concerns, indicating that they were of moderate to high quality ( Figure 3).

### 3.4 Characteristics of included studies

This review included 14 RCTs. Most of the studies had two groups, one experimental and one comparator. Placebo was usually the comparator except in Koban et al. (2017) and Stumpp et al. (2023), where it was employed as the experimental treatment. Unlike other studies, Slavich et al. (2019) had 3 groups; an active group that received acetaminophen, a placebo group that received a pill but it wasn’t acetaminophen, and a control group that did not receive any intervention. The interventions under investigation differed, with most of them being pharmacological except for Keng and Tan (2018) where the treatment group received brief mindfulness and loving-kindness meditation (LKM). Oxytocin ( Alvares, Hickie, & Guastella, 2010; Henningsson et al., 2021; Mohr, Kirsch, & Fotopoulou, 2017; Radke et al., 2020; Zhang et al., 2021) and acetaminophen ( Dewall et al., 2010; Hofman, Wieser, & van der Veen, 2021; Slavich et al., 2019) were the most studied interventions. The largest sample size was 100 in Henningsson et al. (2021), indicating low sample numbers across the studies. The cyberball task was the most used paradigm across studies to induce social exclusion/rejection. Hofman, Wieser, and van der Veen (2021) used a social judgment paradigm (SJP) and Zhang et al. (2021) used online speed dating to induce feelings of romantic rejection.

### 3.5 Effect of acetaminophen

All of the studies on acetaminophen agreed that it led to reduced social pain in cases of social rejection/exclusion as compared to placebo. One of the studies found that a combination of acetaminophen plus forgiveness produced better results compared to acetaminophen or forgiveness alone. Dewall et al. (2010) looked at the neural and behavioral responses of administering acetaminophen. The trial reported reduced reports of social pain and reduced neural responses, from functional magnetic resonance imaging, in brain regions associated with distress caused by social pain (i.e., the dorsal anterior cingulate cortex and anterior insula). Through data analysis and multilevel modeling, it became evident that participants who took acetaminophen experienced a significant decrease in hurt feelings over time, with an average slope of –0.0081 (p < 0.05). In contrast, participants who received a placebo showed no change in their daily hurt feelings over time, with an average slope of 0.0035 (p > 0.55). Additionally, the study noted that the effects of acetaminophen became significant starting on day 9 of daily 1000-mg acetaminophen intake. Hofman, Wieser, and van der Veen (2021) used a social judgment paradigm (SJP) to test the effect of acetaminophen on behavioral response to social rejection. In the control group, there was a decrease in the percentage of acceptance predictions over the course of the experiment, indicating a learning process from negative feedback. However, the Acetaminophen group did not adjust their positive prediction bias over time.

Slavich et al. (2019) reported that over 21 days, regular intake of acetaminophen led to a notable decrease in feelings of social pain. However, this effect was observed only among individuals who demonstrated high levels of forgiveness (i.e., 1 *SD* above the mean). The study confirmed the possibility of an interactive effect between forgiveness and acetaminophen on social pain. It was found that participants with high forgiveness levels experienced significantly greater reductions in social pain over time when they were in the acetaminophen condition compared to those in the placebo-control condition. Participants in the placebo-control and empty-control (no pill) conditions, who scored high in forgiveness (i.e., 1 standard deviation above the mean), reported only slight changes in social pain over time (a decrease of 0.91% and an increase of 1.83% in social pain, respectively). In contrast, participants in the acetaminophen condition, who showed high levels of forgiveness throughout the assessments, experienced an 18.50% decrease in social pain over time. These results suggest a synergistic effect of acetaminophen and forgiveness in reducing social pain. The analysis carried out in the study revealed a significant main effect of forgiveness on changes in social pain over time (F (1, 707.2) = 19.12, p < .0001). This suggests that higher levels of forgiveness on the preceding day were associated with lower levels of social pain on the subsequent day (B = −.104). Furthermore, the results indicated that the impact of forgiveness on next-day social pain levels remained consistent over time.

### 3.6 Effect of oxytocin

Apart from one study, oxytocin was not found to reduce social pain. In Alvares, Hickie, and Guastella (2010) both oxytocin (OT) and placebo-administered participants reported similar levels of social pain during ostracism. Henningsson et al. (2021) noted that oxytocin had a sex, male or female, and context-dependent, being in a romantic relationship, impact on post-ostracism mood ratings. Women who received oxytocin reported experiencing improved mood and a greater sense of inclusion during the cyber ball game, in contrast to women who received a placebo. However, the opposite trend was noted among men. According to Mohr, Kirsch, and Fotopoulou (2017), oxytocin eliminates the connection between neural responses and emotional reactions to social exclusion. Thus, oxytocin was reported not to affect social pain. In Radke et al. (2020), oxytocin appeared to have no effect on how individuals responded to social rejection. Zhang et al. (2021) also looked at the effect of oxytocin. The study used Electroencephalographic recordings and defined frontal-midline theta oscillation as a neural signature of social pain. The theta oscillation in the frontal-midline region appears to originate from brain areas that overlap with the physical-social pain matrix. These areas include the somatosensory cortex, anterior cingulate cortex, frontal pole, and supplementary motor area. Theta oscillation was evaluated in response to romantic rejection in the oxytocin and placebo groups, and differences were compared. In the placebo group, romantic rejection led to increased theta power, which was linked to feelings of distress from rejection. However, this response was not seen in the group that received oxytocin (OT). In the OT group, romantic rejection resulted in decreased theta power compared to the placebo group. Additionally, in the OT group, there was no connection between theta power and feelings of rejection distress *.*

### 3.7 Effect of placebo treatments

In two reported studies, placebo treatment, both deceptive and open label, was found to reduce social pain. Koban et al. (2017) decided to test the effect that a placebo had on reducing physical and social rejection (recent romantic break-up) pain. Participants in the placebo group were informed that the nasal spray was a potent pain reliever that could also alleviate emotional distress and negative feelings. In contrast, participants in the control condition were informed that the spray contained a basic saline solution used to enhance the clarity of fMRI images without any other effects. The research revealed that the placebo treatment effectively diminished feelings of social pain. This was demonstrated by a notable contrast between the Placebo and Control Groups in terms of negative affect before and after treatment (p = 0.001, Cohen’s d = 1.04). Stumpp et al. (2023) discovered that individuals who were excluded and received open-label placebos reported significantly lower levels of hurt feelings than those in the control group (Cohen’s d = 0.77).

### 3.8 Effect of duloxetine and pregabalin

Ghavidel-Parsa et al. (2021) found that duloxetine and pregabalin, which can be used to treat fibromyalgia, did not affect social pain. Of note, these medications were found to be impactful in reducing physical pain and depression but not social pain from invalidation.

### 3.9 Effect of mindfulness training

The study by Keng and Tan (2018) included only adults with Borderline personality disorder (BPD) and used a Hierarchical Linear Modelling (HLM) analysis, to see how 10-minute mindful exercises and meditation would affect the response to social rejection. The study used a no-instruction group as a control. The study found that compared to the control group, the mindfulness group exhibited notably faster recovery in negative mood and feelings of rejection following social rejection.

### 3.10 Effect of glucose

Miller et al. (2014) found that glucose did not have a significant impact on reducing feelings of ostracism or social pain.

### 3.11 Effect of psilocybin

Petereit et al. (2019) reported that participants who received Psilocybin, a psychedelic prodrug compound produced by hundreds of species of fungi, reported a decreased sense of social exclusion compared to those who received a placebo. Additionally, the neural response to social exclusion was reduced in key regions for processing social pain, including the dorsal anterior cingulate cortex (dACC) and the middle frontal gyrus. Given that these brain regions are known to be crucial for processing social exclusion, these findings imply that the administration of Psilocybin decreased the perception of social pain.

## 4. Discussion

This systematic review included 14 RCTs with varying pharmacologic interventions, with the objective of determining which of them leads to a more effective/greater reduction in social pain. Given the complexity of pain management, understanding the relationship between social pain and physical pain has important implications for clinical practice ( Ryans et al., 2024). Healthcare professionals should consider the psychosocial aspects of pain management and incorporate interventions targeting social support and coping strategies into treatment plans for individuals experiencing chronic pain ( Brooks et al., 2023). By addressing both the emotional distress associated with social pain and the physical discomfort of chronic pain, clinicians can provide more holistic care and improve overall well-being for their patients ( Brooks et al., 2023). Additionally, interventions aimed at reducing social stressors and fostering supportive social environments may help mitigate the impact of social pain on physical health outcomes, ultimately leading to better treatment outcomes and enhanced quality of life for individuals dealing with both social and physical pain ( Williams, 2007).

A systematic review with meta-analysis examining the experiences of children and adolescents have shown that early exposure to social rejection and peer victimization can significantly impact health outcomes later in life ( Moore et al., 2017). Psychosocial factors play a significant role in mediating the relationship between social and physical pain. Feelings of loneliness, social isolation, and perceived lack of social support have been associated with increased vulnerability to both social and physical pain ( Kross et al., 2011). Chronic exposure to social stressors, such as bullying or interpersonal conflict, can exacerbate physical pain conditions over time, highlighting the detrimental impact of prolonged social pain on physical well-being ( Hawkley & Cacioppo, 2010). Studies have shown that experiencing social rejection triggers the activation of the body’s stress response system, leading to the release of stress hormones such as cortisol and adrenaline ( MacDonald & Leary, 2005; Rosen et al., 2012; Fairhurst et al., 2012; Chen et al., 2008). These hormones not only modulate emotional responses to social pain but also influence pain perception and sensitivity to physical stimuli ( Zhang, Zhang, & Kong, 2020; Eisenberger, 2015; Kross et al., 2011).

Acetaminophen, both deceptive and open-label placebos, mindfulness training, and psilocybin were found to reduce social pain. However, oxytocin, fibromyalgia drugs (duloxetine and pregabalin), and glucose were reported to have no impact. Understanding the mechanisms behind these outcomes, particularly how the pain relievers were able to reduce social pain, is crucial. The ability of acetaminophen to alleviate social distress is supported by a growing body of literature indicating that certain brain regions are involved in both social and physical pain ( Eisenberger & Lieberman, 2004; Panksepp, 2005). Neuroimaging studies focusing on the emotional or distressing aspects of physical pain often investigate brain regions such as the dorsal anterior cingulate cortex (dACC) and anterior insula ( Apkarian et al., 2005; Peyron, Laurent, & García-Larrea, 2000). Individuals with damage to these brain regions often report a lack of distress from physical pain, despite still being able to feel it ( Berthier, Starkstein, & Leiguarda, 1988). Furthermore, research has shown that these same neural regions are involved in processing experiences of social rejection or loss in humans ( Eisenberger, Lieberman, & Williams, 2003; O’Connor et al., 2008). Given this overlap in neural systems, it is reasonable to assume that factors reducing physical pain would also affect social pain. Studies using acetaminophen, also known as paracetamol, a well-known painkiller, have demonstrated this effect ( Dewall et al., 2010). While the specific mechanisms by which acetaminophen relieves pain are not fully understood, it is generally accepted that acetaminophen acts on the central nervous system rather than the peripheral nervous system ( Anderson, 2008; Smith, 2009). Therefore, acetaminophen may reduce the perception of social pain by reducing neural activity in brain regions involved in both physical and social pain processing, such as the dorsal anterior cingulate cortex (dACC) and the anterior insula.

Hofman, Wieser, and van der Veen (2021) conducted a novel experiment using a social judgment paradigm (SJP) to investigate the effects of acetaminophen on social pain. Participants were either unexpectedly accepted or rejected 15 minutes after taking a single dose of acetaminophen. This approach differed from Dewall et al. (2010), who used a cyberball paradigm and administered acetaminophen over three weeks. Over time, the placebo group reported decreasing expectations of acceptance, indicating learning from negative feedback. In contrast, the acetaminophen group consistently reported similar levels of acceptance. This unexpected impact of acetaminophen on predictive behavior suggests its potential role in altering perceptions of social pain. The results imply that when individuals consume acetaminophen, the typical learning effect related to social pain may disappear, indicating a reduced perception of pain following acetaminophen ingestion.

The study conducted by Slavich et al. (2019) examined how daily forgiveness, when combined with a daily dose of acetaminophen, influenced the level of social pain experienced the following day. The study found that while acetaminophen alone reduced feelings of social pain, those who reported higher levels of forgiveness experienced even greater reductions in social pain. This suggests a new perspective on the impact of acetaminophen in reducing social pain, indicating that a daily combination of acetaminophen and forgiveness is more effective than acetaminophen alone. Importantly, the effectiveness of acetaminophen was still evident, as participants in the placebo group who reported high levels of forgiveness did not experience similar reductions in social pain. These results align with previous research suggesting that both forgiveness and acetaminophen independently reduce the experience of social pain resulting from negative interpersonal interactions ( Akhtar and Barlow, 2016; Dewall et al., 2010). However, this study goes further to demonstrate that the combination of acetaminophen and forgiveness is more effective than either intervention alone.

It has been suggested that the effects of oxytocin (OT) depend heavily on the context ( Declerck et al., 2010). For example, research indicates that OT’s impact on cooperative behavior is only significant when there has been prior social contact ( Alvares, Hickie, & Guastella, 2010). In situations where the interaction partner is anonymous, OT does not seem to have an effect ( Declerck et al., 2010). Ostracism, as a form of social rejection, is a clear and overt social stressor that lacks ambiguity or positive social cues. In Alvares, Hickie, and Guastella (2010), participants engaged in a computer-based task in a laboratory setting where no rewards, social interactions other than with the experimenter, or social encouragement were available to those who were ostracized. In situations where there is a lack of social information or where a social approach is unlikely to lead to a positive outcome, oxytocin does not appear to influence behavior or emotions.

Placebos are therapeutic interventions that, based on the underlying therapeutic theory, are not intended to have any effect on the treated condition ( Grünbaum, 1986). Open-label placebo pills have been found to alleviate symptoms in conditions such as migraine attacks ( Kam-Hansen et al. 2014), irritable bowel syndrome (IBS) ( Kaptchuk et al., 2010), cancer-related fatigue ( Hoenemeyer et al. 2018), and allergic rhinitis ( Schaefer, Harke, & Denke, 2016). Moreover, placebos have been shown to effectively reduce physical pain. Therefore, Koban et al. (2017) and Stumpp et al. (2023) sought to investigate whether placebo pills could also alleviate social pain. Koban et al. (2017) used deceptive placebos, while Stumpp et al. (2023) used open-label placebos, meaning that participants were clearly informed that they were receiving a placebo. Both studies reported positive outcomes, specifically a reduction in social pain. The effects of the placebo can be explained through three proposed mechanisms: pharmacological memory, the influence of a treatment rationale, and “embodied” consciousness ( Colloca & Howick, 2018; Kaptchuk, 2018; Locher et al., 2017). Pharmacological memory suggests that taking any pill, even if known to be a placebo, triggers associations with taking an active drug, leading to a conditioned response ( Colloca & Howick, 2018). The treatment rationale, provided by the experimenter in a friendly and trustworthy manner, can enhance placebo effects by boosting positive expectations ( Locher et al., 2017; Gaab et al., 2019). Embodied cognition theory posits that physical interactions with the world affect cognitions ( Borghi & Caruana, 2015), and placebo effects may arise from bodily sensations shaping pain perceptions and triggering the production of pain-relieving substances ( Colloca & Howick, 2018; Kaptchuk, 2018; Schienle, Unger, & Schwab, 2022). These mechanisms are likely interconnected, with placebo effects depending on the combined influence of all three ( Colloca & Howick, 2018).

The findings of Keng and Tan (2018) suggest that brief mindfulness training accelerates emotional recovery from negative feelings following social rejection, in comparison to a control condition. This is consistent with previous research indicating that brief mindfulness training can alleviate dysphoric mood and negative affect in response to adverse stimuli ( Arch & Craske, 2006; Broderick, 2005; Sauer & Baer, 2012). Keng and Tan (2018) also suggest that mindfulness practice can help moderate emotional responses to social rejection, a stressor that individuals with BPD are particularly sensitive to. Individuals with BPD often engage in avoidance behaviors or excessive rumination on negative thoughts and emotions ( Selby et al., 2009). Mindfulness training was found to assist these individuals in disengaging from avoidance or rumination, enabling them to approach difficult emotions with mindfulness and without judgment, potentially leading to more effective emotion regulation.

The idea that consuming glucose could reduce social pain by enhancing motivation, persistence, and executive control through dopaminergic activity, specifically D1 receptor activation, as suggested by Granon et al. (2000), Touzani, Bodnar, and Sclafani (2010), and Williams (2009), was not supported by Miller et al. (2014). According to their findings, glucose’s rewarding effects rely on D1, not D2, receptor activation in the prefrontal cortex, and D1 agonists can improve executive function. This implies that if D2 activation is necessary to alleviate social pain, glucose may not be effective ( Touzani, Bodnar, & Sclafani, 2010). The lack of social pain relief through D1 activation is consistent with other studies that have explored similar interventions.

Petereit et al. (2019) found that psilocybin administration led to a decrease in activity in the dorsal anterior cingulate cortex (dACC), which did not correspond to a reduction in feelings of social exclusion. This reduced neural response in the dACC was notably associated with changes in self-awareness induced by psilocybin and a decrease in aspartate (Asp) levels. In summary, the stimulation of 5-HT2A/1A receptors with psilocybin appears to reduce the processing of social pain, aligning with changes in self-perception ( Carhart-Harris et al., 2014).

### 4.1 Limitations

Some of the reported research only examined the immediate effects of interventions on social pain. There is a need for more studies to assess the long-term impacts of various interventions. Some studies relied on self-report measures to assess participants’ negative emotions and experiences of rejection. These studies often had small, demographically specific sample sizes, which could affect the generalizability and robustness of their conclusions. Additionally, the interventions, outcome measures, and data analysis techniques varied among studies, leading to heterogeneity. The differences in how quantitative data were reported across studies prevented a meta-analysis from being conducted in this review. Despite these limitations, the conclusions drawn upon by this review are well elaborated and presented. Futures studies examining the impact of social pain as it relates to the Neurogenic Theory of Depression are warranted ( Jacobs et al., 2020).

## 5. Conclusions

Acetaminophen, both deceptive and open-label placebos, mindfulness training, and psilocybin were found to reduce social pain ( Dewall et al., 2010; Hofman, Wieser, & van der Veen, 2021; Keng & Tan, 2018; Koban et al., 2017; Petereit et al., 2019; Preller et al., 2016; Slavich et al., 2019; Stumpp et al., 2023). In contrast to interventions that leverage the neural connection between physical and social pain, mindfulness training focused on developing conscious emotional responses to social exclusion. Additionally, a combination of acetaminophen and forgiveness yielded superior results compared to either acetaminophen or forgiveness alone ( Slavich et al., 2019). However, interventions such as oxytocin, duloxetine, pregabalin, and glucose did not show an impact on social pain ( Alvares, Hickie, & Guastella, 2010; Ghavidel-Parsa et al., 2021; Henningsson et al., 2021; Miller et al., 2014; Mohr, Kirsch, & Fotopoulou, 2017; Radke et al., 2020; Zhang et al., 2021).

## Ethics and consent

Ethics and consent were not required.

## Data Availability

No underlying data are associated with this article. Zenodo repository: Extended data for “Social Pain: A Systematic Review on Interventions” https://doi.org/10.5281/zenodo.14559893 ( Brooks and Brooks, 2024). This project contains following dataset:
1.
Extracted Data for the Systematic Review on the Interventions for Social Pain.xlsx
2.

Figure 1 PRISMA FLOW DIAGRAM for the Systematic Review on Interventions for Social Pain.jpg
3.

Figure 2 Risk of Bias Graph for Included RCTs in the Systematic Review of Social Pain Interventions.jpg
4.

Figure 3 Summary plot of risk of bias of included RCTs in the systematic review.jpg Data are available under the terms of the Creative Commons Zero “No rights reserved” data waiver (CC0 1.0 Public domain dedication). Repository: PRISMA checklist for ‘Social Pain: A Systematic Review on Interventions’. https://doi.org/10.5281/zenodo.14559893 ( Brooks and Brooks, 2024). Data are available under the terms of the Creative Commons Zero “No rights reserved” data waiver (CC0 1.0 Public domain dedication).
