# Supplementary material for: Social pain: A systematic review on interventions
Source: F1000Res. 2025 Jan 10;14:58. [Version 1] doi: 10.12688/f1000research.159561.1 (PMC11937780; doi:10.12688/f1000research.159561.1)
Supplement: Peer review history

# Reviewer report: Reviewer response for version 1

**DOI:** 10.5256/f1000research.175310.r370417

**Published:** 2025-04-09

**Authors:** Cyaandi Dove

**Affiliations:** 1 The University of Texas at San Antonio (UTSA), San Antonio, USA

This is a very timely question, as we see a shift in medicine.  All medical specialties are losing their myopia and have begun looking at the patient is a more holistic manner.  Medical practitioners, in an age of resource scarcity, understand that obtaining optimal health outcomes, depends not just on the medical regimen.  The medical field has not placed a priority on  psychological and social factors, which can have a tremendous impact on treatment and outcomes.  I believe this manuscript raises a very pivotal point and also points to the need for further studies, including interventional studies on this matter.  The manuscript is an important and novel study of social pain, that will beget further, focused work, that can hopefully be translated to bedside medicine.

Are the rationale for, and objectives of, the Systematic Review clearly stated?

Yes

Is the statistical analysis and its interpretation appropriate?

Yes

If this is a Living Systematic Review, is the ‘living’ method appropriate and is the search schedule clearly defined and justified? (‘Living Systematic Review’ or a variation of this term should be included in the title.)

Yes

Are sufficient details of the methods and analysis provided to allow replication by others?

Yes

Are the conclusions drawn adequately supported by the results presented in the review?

Yes

Reviewer Expertise:

Limb Preservation;  Diabetic Foot; Clinical Research

I confirm that I have read this submission and believe that I have an appropriate level of expertise to confirm that it is of an acceptable scientific standard.

# Reviewer report: Reviewer response for version 1

**DOI:** 10.5256/f1000research.175310.r370420

**Published:** 2025-03-25

**Authors:** Qiyang Li

**Affiliations:** 1 New England Sleep Academy, West Second Ring Lake Road, China

**Summary**: The systematic review examines interventions aimed at reducing "social pain" - defined as emotional distress resulting from social exclusion, rejection, or loss. A quite interesting, but unique and thoughtful narrative introduces the concept of using the passion of Christ as an allegorical case of compounded social and physical suffering, through mindfulness and cognitive reframing to forgive and overcome this obstacles.  The review draws on neuroscience and psychological evidence, the authors analyze 14 RCTs to identify both pharmacological and non-pharmacological approaches to mitigate social pain. Notably, acetaminophen, placebos, mindfulness training, and psilocybin showed efficacy, while oxytocin, glucose, and fibromyalgia drugs did not. 

**Evaluation**: Authors clearly articulated the rationale behind studying social pain due to its significant clinical implications. Objectives were explicitly states, targeting identification and summarization of effective interventions. The methodological details, including databases, search terms, including criteria and quality assessment (bias analysis), were comprehensive and clearly outlined according to PRISMA 2020 guidelines (*this enables precise replication).  Due to heterogeneity among those included studies, no meta-analysis or extensive statistical analyses were performed. The authors' qualitative synthesis was comprehensive and well-organized. conclusions were logical, directly supported by the presented results, and appropriately acknowledge study limitations, and interpretations aligned well with the evidence discussed.  

**Strengths**: Well-organized methodology and transparent reporting following PRISMA standards with clear explanation of neurobiological mechanisms underlying social pain interventions. Unique introductory narrative framing using the culturally and historically significant example of Jesus Christ's passion, author effectively conveying the complex human experiences of social rejection, pain, and ultimately forgiveness. 

**Recommendations**: Figure studies should employ longitudinal designs to assess intervention sustainability and effectiveness. Greater exploration into gender-specific or context-dependent responses is recommended. 

**Final Comment**s: This systematic review represents a valuable contribution toward understanding one of the significant daily struggles faced by individuals and society at large. It critically evaluates the effectiveness of existing interventions, both individually and in combination, and identifies promising directions for future approaches. This systematic review effectively bridge neurobiological, psychological, and clinical perspectives, enhancing the depth and applicability of the review. Additionally, incorporating the Passion of Christ narrative enriches the analysis by vividly illustrating the profound human experiences associated with social pain, offering both compelling context and deeper insight into the phenomenon.

Are the rationale for, and objectives of, the Systematic Review clearly stated?

Yes

Is the statistical analysis and its interpretation appropriate?

Not applicable

If this is a Living Systematic Review, is the ‘living’ method appropriate and is the search schedule clearly defined and justified? (‘Living Systematic Review’ or a variation of this term should be included in the title.)

Not applicable

Are sufficient details of the methods and analysis provided to allow replication by others?

Yes

Are the conclusions drawn adequately supported by the results presented in the review?

Yes

Reviewer Expertise:

I hold advanced interdisciplinary academic qualifications, Master of Public Health (MPH), Master of Business Administration (MBA), and Master of Public Administration (MPA), which collectively enable me to evaluate social pain comprehensively from clinical, organizational, and societal perspectives. My MPH training, in particular, equips me with strong methodological expertise, essential for objectively reviewing and critically assessing diverse research studies.In addition, as a Board Director of the Board of Registered Polysomnographic Technologists (BRPT), this role granting me a strategic perspective on healthcare needs, standards, education processes. As a healthcare provider in Sleep Medicine, I have extensive clinical expertise and practical experience in sleep medicine and technology. In my daily clinical practice, I routinely engage with patients who experience social, emotional, and physical pain, deepening my insight into the complex interplay between these factors. This combination of academic and clinical experience uniquely positions me to competently and thoughtfully evaluate research concerning social pain interventions.

I confirm that I have read this submission and believe that I have an appropriate level of expertise to confirm that it is of an acceptable scientific standard.

# Reviewer report: Reviewer response for version 1

**DOI:** 10.5256/f1000research.175310.r370421

**Published:** 2025-03-19

**Authors:** Savannah Santiago

**Affiliations:** 1 Podiatry, Ascension St Vincent Hospital - Indianapolis (Ringgold ID: 24104), Indianapolis, Indiana, USA

Summary of the article: This systematic review explores the relationship between social pain—emotional distress from social rejection, exclusion, or loss—and physical pain. The study highlights how social pain can intensify physical pain, using the Passion of Jesus Christ as a historical example. The review aims to identify interventions that reduce social pain, both pharmacological and non-pharmacological.

 After analyzing 548 articles, 14 randomized controlled trials (RCTs) were included. The key findings indicate that acetaminophen, placebos (both deceptive and open-label), mindfulness training, and psilocybin effectively reduce social pain. Interestingly, the combination of acetaminophen and forgiveness yielded the most significant reduction in social pain. However, oxytocin, glucose, and fibromyalgia medications (duloxetine and pregabalin) did not show a significant impact.

 The mechanisms behind these effects suggest that physical and social pain share overlapping neural pathways, particularly involving the anterior cingulate cortex (ACC) and insula. Acetaminophen appears to dampen the distress response to social rejection, while mindfulness accelerates emotional recovery. Placebos influence expectations and cognitive associations, contributing to pain relief. Psilocybin reduces neural activity in pain-processing regions, altering the perception of social exclusion.

 The study acknowledges limitations, including small sample sizes, reliance on self-reported data, and varying methodologies across studies. More research is needed to assess long-term effects and further explore the link between social pain and mental health conditions like depression.

 Thoughts overall: 

 The use of Jesus Christ was an interesting choice for the introduction but made logical sense and explained the concept well. 

 Figures were clearly labeled and easily interpreted 

 3.10 could of be explained a little further and was fairly brief

Are the rationale for, and objectives of, the Systematic Review clearly stated?

Yes

Is the statistical analysis and its interpretation appropriate?

Yes

If this is a Living Systematic Review, is the ‘living’ method appropriate and is the search schedule clearly defined and justified? (‘Living Systematic Review’ or a variation of this term should be included in the title.)

Not applicable

Are sufficient details of the methods and analysis provided to allow replication by others?

Yes

Are the conclusions drawn adequately supported by the results presented in the review?

Yes

Reviewer Expertise:

Podiatry

I confirm that I have read this submission and believe that I have an appropriate level of expertise to confirm that it is of an acceptable scientific standard.
